# Verloren negatively regulates the expression of IMD pathway dependent antimicrobial peptides in *Drosophila*

**DOI:** 10.1038/s41598-021-94973-0

**Published:** 2021-07-30

**Authors:** Pragya Prakash, Arghyashree Roychowdhury-Sinha, Akira Goto

**Affiliations:** 1 INSERM, Université de Strasbourg, CNRS, Insect Models of Innate Immunity (M3I; UPR9022), 67084 Strasbourg, France; 2 Sino-French Hoffmann Institute, School of Basic Medical Science, Guangzhou Medical University, Guangzhou, 511436 China

**Keywords:** DNA, Immunochemistry, RNA, Biochemistry, Genetics, Gene expression, Gene regulation, RNAi, Immunology, Infection, Inflammation, Innate immune cells, Innate immunity, Signal transduction, Molecular biology, RNAi, Transcription, Microbiology, Bacteria, Fungi, Pathogens

## Abstract

*Drosophila* immune deficiency (IMD) pathway is similar to the human tumor necrosis factor receptor (TNFR) signaling pathway and is preferentially activated by Gram-negative bacterial infection. Recent studies highlighted the importance of IMD pathway regulation as it is tightly controlled by numbers of negative regulators at multiple levels. Here, we report a new negative regulator of the IMD pathway, Verloren (Velo). Silencing of *Velo* led to constitutive expression of the IMD pathway dependent antimicrobial peptides (AMPs), and *Escherichia coli* stimulation further enhanced the AMP expression. Epistatic analysis indicated that *Velo* knock-down mediated AMP upregulation is dependent on the canonical members of the IMD pathway. The immune fluorescent study using overexpression constructs revealed that Velo resides both in the nucleus and cytoplasm, but the majority (~ 75%) is localized in the nucleus. We also observed from in vivo analysis that *Velo* knock-down flies exhibit significant upregulation of the AMP expression and reduced bacterial load. Survival experiments showed that *Velo* knock-down flies have a short lifespan and are susceptible to the infection of pathogenic Gram-negative bacteria, *P. aeruginosa*. Taken together, these data suggest that Velo is an additional new negative regulator of the IMD pathway, possibly acting in both the nucleus and cytoplasm.

## Introduction

Innate immunity provides a potent host defense against microbial infections. A plethora of genetic tools, less genetic redundancy, and most importantly the lack of adaptive immunity and a high degree of evolutionary conservation to mammals have made the fruit fly *Drosophila melanogaster* an excellent model organism to decipher the fundamental molecular mechanism of innate immunity^1–5^. The *Drosophila* innate immunity is mainly composed of two systems, namely cellular and humoral immune responses. The cellular immune reaction is mediated by insect blood cells called hemocytes involving in various cellular aspects such as phagocytosis, encapsulation, melanization, etc. The humoral response is characterized by the challenge-induced transcription of hundreds of immune response effectors, including antimicrobial peptides (AMPs), predominantly in the fat body (an insect equivalent of the mammalian liver), the hemocytes, and barrier epithelia, namely in the gut and the tracheal systems. The AMPs are positively charged amphipathic small peptides. They are classically known to eliminate invading microorganisms generally by using two mechanisms: (1) membrane disruption and 2) inhibition of essential cellular functions^6–9^. However, recent findings revealed that the function of AMPs is not only restricted to microbe killing but also extended to other biological aspects such as gut homeostasis, neurology, and tumor control^10^. There are two distinct innate immune signaling pathways known in *Drosophila*, namely the Toll and immune deficiency (IMD) pathways^11,12^. They share similarity to mammalian Toll-like receptor signaling pathway and tumor necrosis factor α receptor (TNFR) signaling pathway, respectively and control the expression of respective AMP readouts such as *Drosomycin* and *Diptericin*^13,14^. The Toll pathway is mainly activated by fungal and/or Gram-positive bacteria and mediated by several factors such as Toll receptor, *Drosophila* myeloid differentiation primary response 88 (dMyD88)^15^, tube and pelle kinase^16^, and dorsal related immunity factor (Dif)^17^. Sensing and recognition of pathogen-associated molecular patterns (PAMPs) and of the so-called danger signals in the Toll pathway are mediated through secreted receptors such as β-glucan binding protein 1 and 3 (GNBP-1, -3), peptidoglycan recognition protein short-type A (PGRP-SA)^18–20^, and Psh^21,22^. In contrast, the IMD pathway is preferentially activated by Gram-negative bacteria and initiated by the direct binding of the diaminopimelic acid-type peptidoglycan (DAP-type PGN), which is common to most Gram-negative bacteria, to the peptidoglycan recognition protein long-type C (PGRP-LC) and PGRP-LE receptors^23–25^. Mechanistically, this ligand-receptor binding induces the adaptor complex composed of death domain containing IMD^12,26^, Fas-associated death domain (FADD)^27,28^, and Death related ced-3/Nedd2-like caspase (DREDD)^29^. DREDD becomes activated with ubiquitination by Death-associated inhibitor of apoptosis 2 (DIAP2)^30–32^. Activated Dredd cleaves IMD and leads to the activation of TGF-β activated kinase 1 (TAK1)^33^/TAK1-associated binding protein 2 (TAB2)^31^ complex. The dTAK1/dTAB2 complex is responsible to activate the IKK signalosome composing of dIKKβ (ird5)^34^ and dIKKγ (key)^35^, which in turn cleaves the NF-κB protein Relish. The activated transcription factor Relish is then delivered into the nucleus and triggers hundreds of effectors, including AMPs such as *Diptericin*^34,36,37^.

Flies deficient for the above mentioned positive regulators lead to compromised immune response, resulting in bacterial overload and high susceptibility to infection^13,14^. Notably, flies deficient for negative regulators also die quickly in association with enhanced activation of the IMD pathway^38,39^. It is generally considered that an uncontrolled immune system leads to detrimental effects in the host, but the underlying mechanism remains elusive. In the IMD pathway, several negative regulators have been identified. PGRP-SC and PGRP-LB amidases degrade PGN into non-stimulatory fragments outside of the cell^40,41^. PGRP-LF interacts with PGRP-LC and produces inactive PGRP-LF/PGRP-LC heterodimer^42^. Inside the cell, Pirk interacts with IMD to disrupt the receptor complex^43–45^. Interestingly, Ragab et al. further discovered that the induction of Pirk occurs through the activation of PVR/Ras-MAPK pathway^46^. The ubiquitin-specific protease, dUSP36/Scny, and deubiquitinating enzyme, faf, are involved in the degradation of ubiquitinated IMD^47,48^. The E3 ubiquitin ligase, Plenty of SH3s (POSH), was shown to poly-ubiquitinate TAK1 for the proteasomal degradation^49^. Trabid and Cylindromatosis (CYLD) deubiquitinases bind to dTAK1^50^ and dIKKγ (key)^51,52^, respectively and modulate K63-linked polyubiquitination of their respective target. Ubiquitin binding protein dRYBP was proposed to function for the degradation of Relish together with SKPA^53^. As such, a number of negative regulators were shown to participate in the process of ubiquitination/de-ubiquitination in the IMD pathway.

To further advance the knowledge, Fukuyama et al. undertook a pathway-wide and time-lapse proteomic analysis using 11 canonical components of the IMD pathway and identified ~ 400 interacting proteins^54^. Following RNAi-mediated knock-down experiment, more than half of the candidates show a dysregulated expression of the *Attacin A-luciferase* reporter, proving that the system is potent enough to identify promising candidates. Gene ontology analysis of candidates revealed a significant signature of “small ubiquitin-like modifier (SUMO) binding” as one of the important molecular functions. Indeed, flies heterozygote for the gene encoding E2 SUMO conjugating enzyme, DmUbc9 (*lesswright* mutant: *lwr*), were highly susceptible to *Escherichia coli* (*E. coli*) infection. The *lwr* flies had an increased bacterial burden and showed a reduced expression of *Attacin A*, a representative AMP of the IMD pathway. Further biochemical experiments provided evidence that one of the components of the IMD pathway namely dIKKβ is SUMOylated upon heat-killed *E. coli* stimulation. These results suggest that the SUMOylation plays a critical role in the activation of the IMD pathway^54^.

In this study, we focused on Verloren (Velo), which encodes a SUMO-specific protease and was originally identified from the IMD pathway interactome. We first observed that silencing of *Velo* led to upregulation of the AMP expression both in S2 cells and adult flies. Epistasis analysis showed that this AMP upregulation by knocking down of *Velo* depends on the canonical components of the IMD pathway, however, overexpression of Velo did not repress the IMD pathway. Cellular localization study indicated that the majority of Velo is localized in the nucleus, but some are also in the cytoplasm. *Velo* knock-down flies also showed upregulation of the AMP expression, but they had reduced lifespan and were susceptible to pathogenic Gram-negative bacteria *P. aeruginosa* infection. Taken together, we propose that Velo is a new negative regulator, which controls the expression of IMD pathway dependent AMPs.

## Materials and methods

### Fly strains

Stocks were raised on standard cornmeal–yeast–agar medium (63 g of cornmeal, 11 g of yeast powder, 4.8 g of agar, 47 g of sugar and 4.2 g of methylhydroxyl-4 benzoate per litter) at 25 °C with 60% relative humidity. Transgenic flies for *Velo* knock-down (VDRC ID: 18004-GD and 103524-KK; carrying a UAS-RNAi against Verloren) and *dIKKβ* (*ird5*) knock-down (VDRC ID: 26427; carrying a UAS-RNAi against *dIKKβ* (*ird5*)) were obtained from the Vienna *Drosophila* RNAi Center (VDRC). As controls, we used flies carrying UAS-RNAi transgene against *GFP* (397-05) obtained from Kyoto stock center (DGRC) or VDRC (VDRC ID: 60100). *Relish*^*E*^^20^ and *Dif*^*nmc*^^55^ were used as a mutant deficient for the IMD and Toll pathway, respectively. Flies carrying Gal4 drivers, ubiquitous *daughterless* (*da*-Gal4) and the fat body-specific (*c564*-Gal4), were obtained from Bloomington *Drosophila* Stock Center (Bloomington, USA).

### Microbial strains and infections

The entomopathogenic fungi *B. bassiana* was used to perform natural fungal infection^18^. Pathogenic Gram-negative bacterial strain *P. aeruginosa*^56^ was used for septic injuries. The survival experiment was performed by pricking the flies with a sharp tungsten needle dipped in the concentration of OD_600_ = 0.5 bacterial solution. Sterile injury with PBS was used as a control. After the infection, the survival of the flies was monitored over 11 days at 25 °C.

### Colony forming unit (CFU) assay

Flies were pricked with a concentrated kanamycin-resistant *E. coli* MC4100 strain carrying the pBB2:GFP plasmid (a gift from Dr. Eleonora García Véscovi) or OD_600_ = 1.0 *P. aeruginosa* solution. After 6 h or 24 h incubation of the flies at 29 °C, a total of 5 flies per sample with at least 8 biological replicates for each genotype were homogenized in 150 μl of LB medium, serially diluted, and plated onto the kanamycin containing LB agar plates. The next day, numbers of colonies were counted to calculate CFU per fly.

### Quantitative RT-qPCR

For quantitative expression analysis of *Diptericin*, *Attacin*, *Metchnikowin*, *Cecropin*, *Drosomycin,* and *Velo*, total RNAs from the flies were isolated by the standard protocol using a TRIzol Reagent RT bromoanisole solution (MRC). Briefly, 1 µg of total RNA was reverse transcribed using iScript TM cDNA synthesis Kit (Biorad). Real-time PCR was performed using 100 ng of cDNAs in 384-well plates on CFX384 Touch™ Real-Time PCR Detection System (Biorad). Normalization was performed with the housekeeping gene *Ribosomal protein 49* (*Rp49*). The qPCR data were analyzed by the ΔΔCT method. Sequences of RT-qPCR primers are shown in Supplementary Table 1.

### Synthesis of double-stranded (ds) RNAs

The PCR fragments with two T7 promoter sequences at both ends were amplified by PCR and used for the templates for dsRNA production. Fragments for each gene were as follows: *GFP* (nt 26–302, GenBank L29345), *Velo* (nt 1540–1881 and 1933–2266, NCBI NM_139799), *Imd* (nt 146–490, NCBI NM_133166), *PGRP-LC* (nt 365–620, NCBI NM_001169925), *Relish* (nt 848–1107, NCBI NM_057746). dsRNAs were synthesized by in vitro transcription with T7 MEGAscript T7 transcription kit (AM1334; Ambion). Two independent dsRNAs for *Velo* were generated to eliminate the possibility of an off-target effect. dsRNA against *GFP* (*dsGFP*) was used as a negative control.

### Cell culture and transfection

Schneider 2 (S2) cells were cultured at 25 °C in Schneider's medium (Biowest) supplemented with 10% fetal calf serum (FCS) and 8 mM penicillin/streptomycin (Gibco). For transient transfection, a total of 0.6 × 10^6^ cells were seeded per well in a 24-well plate. Transfection was performed by the calcium phosphate co-precipitation method. Each plate was co-transfected with 1 μg of indicated tagged overexpression plasmids (pMT-short Velo-V5, pAC-long Velo-WT-HA^57^, pAC-long Velo-CS-HA^57^, pAC-PGRP-LC (TM + Intra)-V5^58^, or pAC-RelishΔS29-S45^36^), 50 ng of *AttacinA-firefly luciferase* reporter, 10 ng of *Actin5C-renilla luciferase* transfection control reporter, and each dsRNAs (2.0 μg/well). After 12-16 h of the transfection, the cells were washed with PBS and incubated in a fresh medium with or without 500 μM CuSO_4_. The next day, cells were stimulated with heat-killed *E. coli* for 24 h with the multiplicity of infection (MOI) = 40. Firefly and Renilla luciferase activities of the cell lysate were measured by a dual luciferase assay kit (Promega).

### Immunoprecipitation and western blot

After transfection of the indicated plasmids at 72 h, the cells were harvested, washed by PBS, and lysed in lysis buffer containing a complete protease inhibitor cocktail (Roche). Immunoprecipitation was performed overnight with rotation at 4 °C, using monoclonal anti-V5 antibody coupled to agarose beads (Sigma, A7345). Proteins from immune-precipitates and total cell lysates were separated by SDS-PAGE and detected by western blotting using rabbit anti-HA (1;3000; Abcam, ab9110)/mouse anti-HA (1:3000; Roche, 12CA5, 11583816001), rabbit anti-V5 (1:3000; Abcam, ab9116), and/or mouse anti-actin (1:10,000; Millipore, clone C4, MAB1501R) antibodies.

### Immunofluorescence

Cells were seeded on 8-wells Lab-Tek^®^ Chamber Slide, washed with PBS, and fixed with 2% paraformaldehyde. Cells were then permeabilized with 0.1% Triton X-100 and blocked with bovine serum albumin (BSA). After blocking, samples were incubated with mouse anti-HA (1:500) or mouse anti-V5 (1:500) antibody overnight at 4 °C. After washing cells, Alexa 488 anti-mouse (Thermo Fischer Scientific, A28175) was used for the secondary antibody reaction with 1:500 dilution. Slides were mounted in Vectashield/DAPI solution and samples were imaged using a Zeiss LSM780 confocal microscope. Images were subsequently processed using ImageJ or Photoshop software.

### Statistical analysis

Unpaired two-tailed Student's *t* test was used for statistical analysis of data with GraphPad Prism (GraphPad Software). Error bars indicate standard deviation. Survival curves were plotted and analyed by log-rank analysis (Kaplan–Meier method) using GraphPad Prism (GraphPad Software). *p* values lower than 0.05 were considered statistically significant.

## Results

### *Velo* knock-down upregulates expression of the IMD pathway regulated AMPs

**Figure 1:**
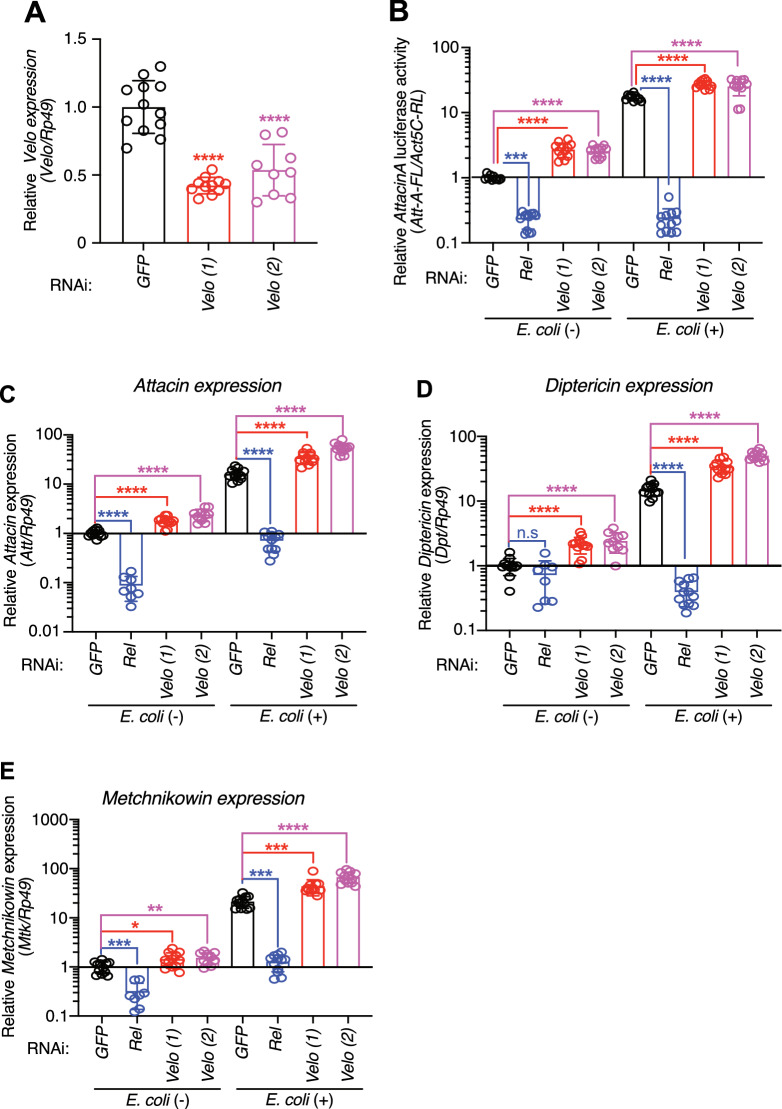
Upregulation of the IMD pathway-regulated AMPs in *Velo* knock-down cells. (**A**) Two dsRNAs targeting to different regions of *Velo* transcript (designated as *Velo(1)* and *Velo(2)*) were synthesized to detect a possible off-target effect. dsRNA against *GFP* was used as a negative control. Three days after the dsRNA transfection into S2 cells, total RNAs were extracted from the cells and *Velo* expression was monitored by RT-qPCR. Expression of the Ribosomal protein 49 (*Rp49*) was used as the internal control for normalization. (**B**) S2 cells were transfected with the indicated dsRNA together with the *AttacinA-luciferase* (*Att-A-FL*) and the transfection control *Actin5C-Renilla luciferase* (*Act5C-RL*) reporters. After stimulation with heat-killed *E. coli* (40 bacteria/cell), the relative *AttacinA-luciferase* activity was calculated based on the ratio of *Att-A-FL/Act5C-RL*. The value of control *GFP* knock-down cells was set as 1. dsRNAs against *GFP* and *Relish (Rel)* were used as a negative or positive control, respectively. (**C–E**) Same as in (**B**), S2 cells were transfected with the indicated dsRNAs, and endogenous expression level of antimicrobial peptides (AMPs) namely *Attacin* (**C**), *Diptericin* (**D**), and *Metchnikowin* (**E**) was monitored by RT-qPCR. Expression of the *Rp49* was used as the internal control for normalization. The data points were collected from three independent experiments. Each experiment includes at least two bio-replicates. Student’s *t* test was used for statistical analysis: *p < 0.05, **p < 0.01, ***p < 0.001, ****p < 0.0001. *n.s.* indicates statistically non-significant.

Our preliminary RNAi screen of the IMD pathway interactome candidates indicated Verloren (*Velo*, CG10107), a putative SUMO specific protease, as a negative regulator of the IMD pathway. We first confirmed by using two independent dsRNAs that *Velo* is significantly knocked down (Fig. 1A). We next monitored activation of the IMD pathway by using *Attacin-luciferase* reporter in the absence or presence of heat-killed *E. coli* (HKE). Consistent with the preliminary RNAi screen result, the knock-down of *Velo* resulted in upregulation of *Attacin-luciferase* activity both in the absence and presence of HKE stimulation (Fig. 1B). We observed a similar upregulation of endogenous AMPs, namely *Attacin*, *Diptericin,* and *Metchnikowin* (Fig. 1C–E), further confirming that *Velo* knock-down triggers the induction of the IMD pathway dependent antimicrobial peptide expression at both basal and immune-stimulated conditions.

### Upregulation of AMP expression by *Velo* knock-down is dependent on the canonical components of the IMD pathway

**Figure 2:**
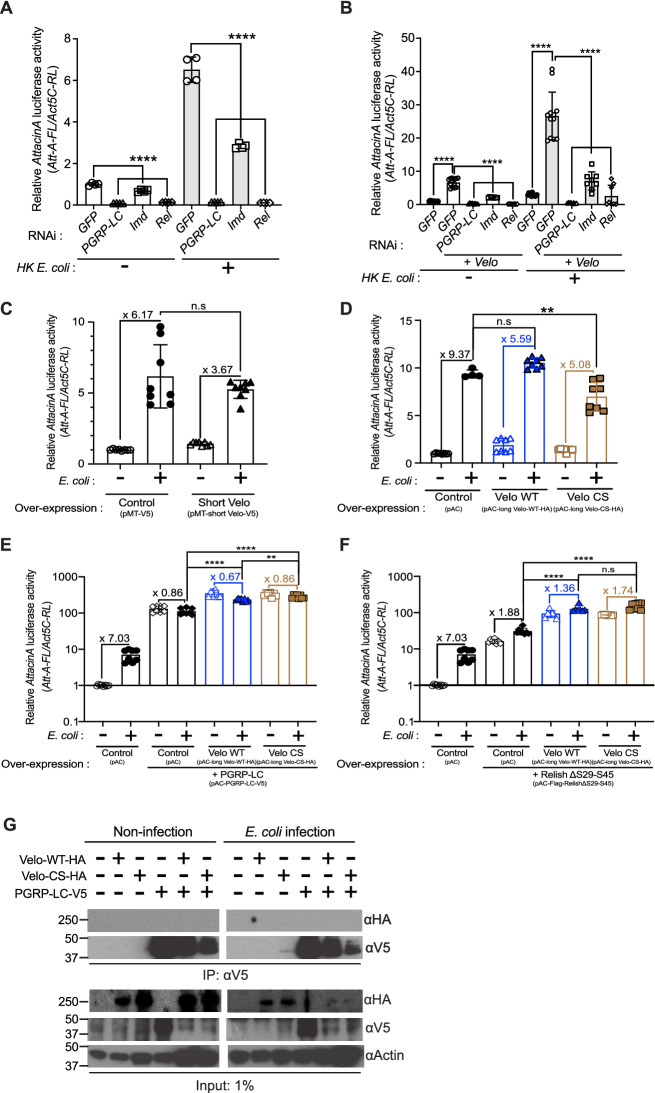
Epistatic analyses of Velo in the IMD pathway. (**A**,**B**) *PGRP-LC*, *IMD,* or *Rel*, was knocked down individually (**A**) or together with *Velo* (**B**). After the indicated dsRNA transfection, the activation of the IMD pathway was monitored by *AttacinA-luciferase* activity (*Att-A-FL/Act5C-RL*) in the absence or presence of heat-killed *E. coli* stimulation. The value of *GFP* knock-down control cells was set as 1. (**C**,**D**) After transfection of the plasmids encoding for a short-form (Short Velo) (**C**) or long-form of Velos (either wild-type “Velo WT” or protease-dead “Velo CS”) (**D**), the *AttacinA-luciferase* activity was measured in the absence or presence of heat-killed *E. coli* stimulation. (**E**,**F**) Long-form of Velo (Velo WT-HA or Velo CS-HA) was co-expressed either with PGRP-LC (**E**) or Relish (**F**), and the *AttacinA-luciferase* activity was measured. The data points were collected from two independent experiments. Each experiment includes at least two bio-replicates. Student’s *t*-test was used for statistical analysis: **p < 0.01, ****p < 0.0001. n.s. indicates statistically non-significant. (**G**) S2 cells were co-transfected with plasmids encoding for the tagged PGRP-LC (PGRP-LC-V5) and long-form of Velo (Velo-WT-HA or Velo-CS-HA). After stimulation of the transfected cells by heat-killed *E. coli*, the cell lysates were co-immunoprecipitated (IP) and immunoblotted by the indicated antibodies. Empty vector transfection and single overexpression of PGRP-LC or Velo were used as controls. 1% of the volume of cell lysate was used as input.

To investigate the cellular target of Velo in the IMD pathway, we next performed the epistatic analysis by using double knock-down of *Velo* and the canonical components of the IMD pathway (Fig. 2). We chose three major components of the pathway, namely PGRP-LC (recognition), IMD (adaptor), or Relish (transcription factor). We first confirmed that the knock-down of each gene significantly represses *Attacin-luciferase* reporter activation (Fig. 2A). We then examined the effect of *Velo* by double knock-down experiment. The result showed that constitutive activation of *AttacinA-luciferase* activity by *Velo* knock-down was significantly repressed in all three gene knock-down conditions. This repression was also observed in the presence of HKE stimulation (Fig. 2B). Of note, a similar result was observed when other components of the pathway namely DIAP2, TAB2, or IKKβ was knocked down (Supplementary Fig. 1).

We next investigated if overexpression of Velo modulates the IMD pathway signaling. There are two isoforms of Velo, namely short-form (711 aa) and long-form (1,833 aa). The short-form lacks the N-terminal Glutamine-rich region. We constructed copper-inducible pMT plasmid expressing a V5-tagged short-form of Velo and used HA-tagged long-forms of Velo, namely Velo-WT (wild-type) and Velo-CS (point mutation from cysteine to serine at amino acid position 1,624) (gift from Pr. Liqun Luo)^57^. We confirmed by RT-qPCR that *Velo* is overexpressed (~ 600-fold for the short-form and ~ 100-fold for the long-form) (Supplementary Fig. 2A,B). However, we did not observe any significant effect on the induction of *Attacin-luciferase* activity (Fig. 2C,D). A similar result was noted for the endogenous expression of *Attacin* and *Cecropin* (Supplementary Fig. 2C–F). We also tried to overexpress Velo in the context of PGRP-LC or Relish overexpression where the IMD pathway becomes constitutively active. In both cases, we did not observe any significant repression of the *Attacin-luciferase* activity (Fig. 2E,F).

Although the overexpression of Velo did not impact the IMD pathway, the result of double knock-down partially suggested that Velo may act at parallel or upstream of PGRP-LC (Fig. 2B). We, therefore, tested the interaction between Velo and PGRP-LC by co-immunoprecipitation assay. While PGRP-LC-V5 was detected with an anti-V5 antibody, the band corresponding to Velo in the complex was not detected by the anti-HA antibody (Fig. 2G). Therefore, we did not observe the interaction between PGRP-LC and Velo in our experimental condition.

### Velo is mostly localized in the nucleus but some are in the cytoplasm

**Figure 3:**
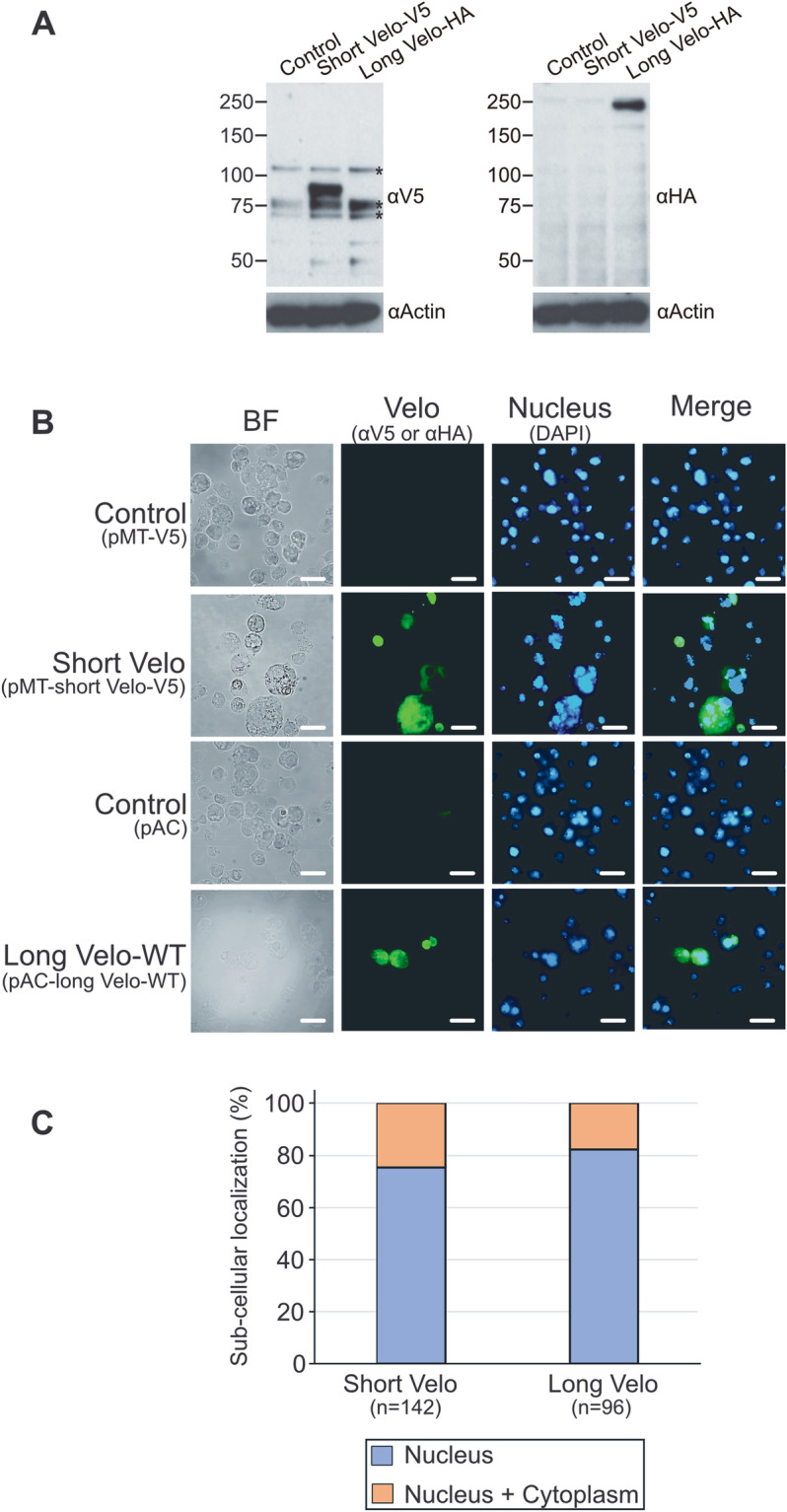
Velo localizes mostly in the nucleus but also in the cytosol. (**A**) S2 cells were transfected with the plasmid expressing either short- or long-form of Velo (designated as Short Velo and Long Velo-WT, respectively). For Short Velo in which *Velo* cDNA is inserted in the metallothionein driven pMT vector, the transfected cells were harvested 24 h after the CuSO_4_ induction. For Long Velo-WT constructed in *Actin5C*-promoter driven pAC vector, the cells were collected 48 h after the transfection. Cell lysates were immunoblotted with indicated antibodies. Detection of actin was used as an internal loading control. Asterisks indicate non-specific bands, which were detected in control empty vector transfected cells (mock). (**B**) After the transfection, the cells were stained by anti-V5 (for short Velo) or anti-HA antibody (for long Velo-WT) and revealed by Alexa488 (green) secondary antibody. DAPI (blue) was used to stain the nucleus. Bar indicates 10 μm. (**C**) The numbers of stained cells indicated were analyzed for the subcellular localization of Velo either in the nucleus or the nucleus + cytoplasm.

We confirmed by western blot that tagged short- and long-form of Velos (short Velo-V5 and long Velo-HA) are expressed at the expected size, ~ 81-kDa and ~ 204-kDa, respectively (Fig. 3A). We next examined the cellular localization of Velo by immune cell staining. The result showed that both forms are localized in the nucleus and cytoplasm (Fig. 3B). After counting the numbers of stained cells, we noted that the majority of Velo localizes in the nucleus (~ 75%). In addition, we found that the short-form is more diffused into the cytoplasm (~ 25.0% of Velo short-form localizes in the cytoplasm, whereas it was ~ 17.7% for the long Velo-WT) (Fig. 3C and see discussion). The cytoplasmic localization of long Velo-CS was similar to that of long Velo-WT (~ 17.6%). Although we do not exclude the possibility that ectopically overexpressed Velo modifies the subcellular localization, these results suggest that the majority of Velo especially the long-form is localized in the nucleus (see also in “Discussion”).

### Effect of Velo knock-down in in vivo adult flies

**Figure 4:**
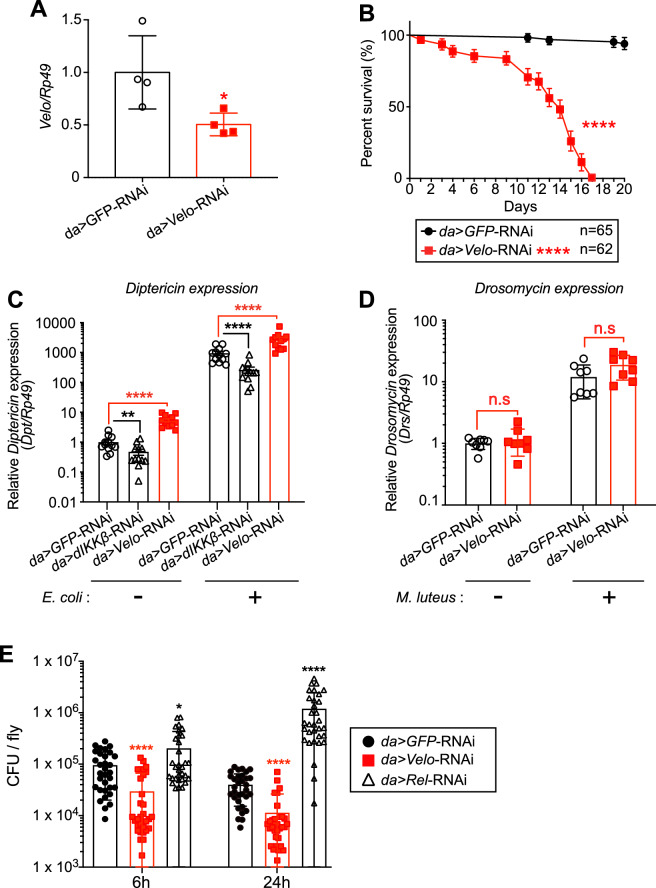
Ubiquitous knock-down of *Velo* results in shortened lifespan, elevated *Diptericin* expression, and decreased injected *E. coli* bacterial load in adult flies. (**A**) Ubiquitous *Velo* knock-down flies were generated by crossing UAS-*Velo*-RNAi with *daughterless(da)-GAL4* line at 25 °C (*da* > *Velo-*RNAi). The knock-down efficiency of *Velo* expression was examined by RT-qPCR. *da* > *GFP*-RNAi flies were used as a negative control. Expression of the Ribosomal protein 49 (*Rp49*) was used as the internal control for normalization. (**B**) The lifespan of *da* > *Velo-RNAi* flies was analyzed by counting the numbers of surviving flies daily at 25 °C. Data represent the mean with at least six biological replicates. The log-rank test was used to calculate the significance of survival curves for statistical analysis. (**C**,**D**) Three to five days after the eclosion, *da* > *Velo-RNAi* flies were pricked either with *E. coli* or *M. luteus* and the expression of *Diptericin* (**C**) or *Drosomycin* (**D**) were monitored at 6 h or 24 h post-infection, respectively. *da* > *dIKKβ-RNAi* flies were used as a positive control. (**E**) *da* > *Velo*-RNAi flies were infected by kanamycin-resistant *E. coli*, and the bacterial load was monitored at indicated time points. The data represent the mean and standard error of three independent experiments, and one data point represents a pool of 5–8 flies. *da* > *Rel-RNAi* flies were used as a positive control. The difference between control *GFP* and each target RNAi is statistically significant (student’s *t* test: *p < 0.05, ****p < 0.0001).

To investigate the in vivo function of *Velo*, we generated *Velo* knock-down flies by crossing the UAS-*Velo*-RNAi line with ubiquitous *daughterless* (*da)-Gal4* driver (hereafter referred to as *da* > *Velo*-RNAi flies). We first noted that many of *da* > *Velo*-RNAi flies died out during larval/pupal stages, pointing that strong knock-down of *Velo* induces a developmental defect as previously reported^57^. Consistently, *da* > *Velo* RNAi adult escapers showed a marginal 50% knock-down efficiency (Fig. 4A) and led to the demise after 17 days (Fig. 4B). Nonetheless, *da* > *Velo* RNAi escapers showed an elevated level of *Diptericin* expression at both basal and *E. coli* stimulated conditions (Fig. 4C). To examine the direct impact of the injected bacteria, we performed a colony formation unit (CFU) assay. The result showed that *da* > *Velo*-RNAi escapers reduced injected *E. coli* burden (Fig. 4E). Interestingly, the expression of the Toll pathway readout namely *Drosomycin* was unaffected upon the infection of *Micrococcus luteus* (Fig. 4D). These results indicate that Velo is specifically required for the downregulation of the IMD pathway both in vitro and in vivo.

### *Velo* knock-down flies are susceptible to the infection of Gram-negative bacteria *P. aeruginosa* but not to fungi *B. bassiana*

**Figure 5:**
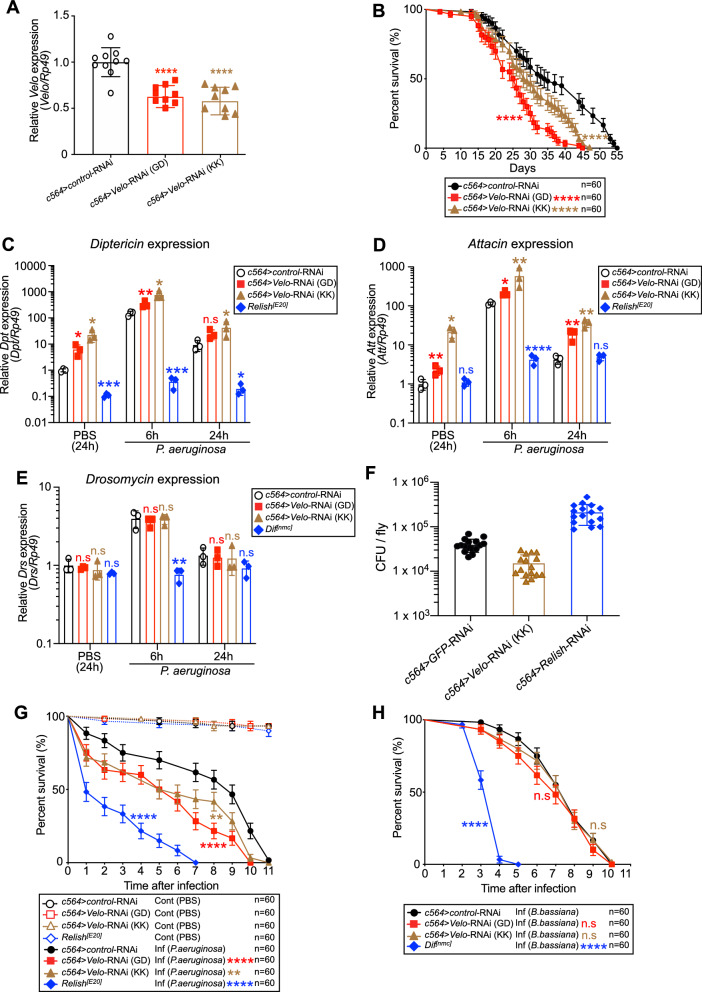
Fat body-specific *Velo* knock-down flies show a moderate lifespan defect, elevated expression of the IMD pathway controlled AMPs, and high susceptibility to *P. aeruginosa* infection. (**A**) Two independent UAS-*Velo*-RNAi flies designated as (GD) and (KK) were crossed with *c564-GAL4* driver to generate the fat body-specific *Velo* knock-down flies (*c564* > *Velo*-RNAi). The level of *Velo* expression was measured as compared to that of control flies (*c564* > *control*-RNAi). Expression of the Ribosomal protein 49 (*Rp49*) was used as the internal control for normalization. (**B**) The lifespan of two independent *c564* > *Velo-RNAi* lines, as well as its control-RNAi, was monitored by counting the numbers of surviving flies daily at 25 °C. (**C**–**E**) *c564* > *Velo-RNAi* flies were pricked by *P. aeruginosa* and the expression of *Diptericin, Attacin, and Drosomycin* were monitored at 6 h and 24 h post-infection. (**F**) Twenty-four hour after infection of *P. aeruginosa*, the bacterial burden on *c564* > *Velo-RNAi* flies was monitored by CFU assay. The data represent the mean and standard error of three biological replicates, and one data point represents a pool of 6–8 flies. The difference between control-RNAi and each *Velo*-RNAi is statistically significant (student’s *t* test: *p < 0.05, **p < 0.01, ***p < 0.001. n.s. indicates statistically non-significant). (**G**,**H**) The survival of the flies after infection of *P. aeruginosa* (**G**) or naturally with *B. bassiana* (**H**) was monitored. Control PBS buffer pricked flies were used as control. *Relish*^*[E20]*^ or *Dif*^*[nmc]*^ null mutant flies were used as positive controls. Data represent the mean with at least six biological replicates. The log-rank test was used to calculate the significance of survival curves for statistical analysis.

Partial lethality at larval/pupal stage and short lifespan of *da* > *Velo*-RNAi adult escapers made it difficult to pursue investigating the in vivo function of Velo in response to microbial infections. We, therefore, generated flies which has *Velo* knocked down only in major innate immune organs, namely the fat body using the *c564-Gal4* driver. We confirmed that *Velo* is significantly knocked down in two different lines of *c564* > *Velo*-RNAi flies (designated as GD and KK) (Fig. 5A). The lifespan experiment showed that both lines had a shorter lifespan as compared to control RNAi flies (Fig. 5B). Nevertheless, *c564* > *Velo*-RNAi flies survived far longer than *da* > *Velo*-RNAi escapers (45 days for *c564* > *Velo*-RNAi vs 17 days for *da* > *Velo*-RNAi) and they did not show any compromised survival effect at least until 15 days after emerging. We, therefore, used ~ 3 to 5 day-old *c564* > *Velo*-RNAi flies in the following infection experiment. As shown in Fig. 5C,D, RT-qPCR result showed that the expression level of the IMD pathway-regulated AMPs, namely *Diptericin* and *Attacin,* was significantly increased upon infection of *P. aeruginosa*, whereas *Drosomycin* and *Puckered* expressions were unaffected (Fig. 5E, Supplementary Fig. 3). Consistent with Fig. 4E, CFU assay indicated that *c564* > *Velo*-RNAi flies had a reduced *P. aeruginosa* burden (Fig. 5F). Nevertheless, *c564* > *Velo*-RNAi flies were highly susceptible to *P. aeruginosa* infection (Fig. 5G)*.* In contrast, they did not show any obvious effect after the infection of *B. bassiana* (Fig. 5H). These results confirmed that Velo negatively regulates the IMD pathway at major immune organs, namely the fat body in adult flies.

## Discussion

*Verloren* (*Velo*) means “Loss” in Germany as this gene was originally named after the lethal phenotype with a severe loss of neuronal pathfinding in embryos^57^. Indeed, previous RNAi screens had scored Velo as an essential gene involved in various cellular processes and signaling pathways such as (1) cell growth and viability^59^, (2) stem cell maintenance^60^, (3) Notch pathway regulation^61,62^, (4) Wnt signaling^63^, etc. These data indicate that Velo is involved in diverse developmental processes. Consistently, we observed that knock-down of *Velo* in flies led to a partial lethality at larval/pupal stage (with *da-Gal4* driver), reduced lifespan (with both *da-Gal4* and *c564-Gal4* drivers) and high susceptibility to *P. aeruginosa* infection. Nonetheless, both *da* > *Velo*-RNAi and *c564* > *Velo-*RNAi flies showed the upregulation of the IMD pathway dependent AMP expression. Given the facts that fly mutants for negative regulators of the IMD pathway are reported to have a short lifespan and some displayed high susceptibility to bacterial infections^40,64,65^, our result suggests that the high lethality of *c564* > *Velo*-RNAi flies is due to an excess activation of the IMD pathway upon bacterial infection. Importantly, this AMP upregulation was also observed in S2 cells specific to the IMD pathway but not to the Toll pathway. In this context, our data is the first demonstration that Velo is involved not only in development but also in the innate immune response by negative regulation of the IMD pathway.

To address where Velo acts in the IMD pathway, we performed two experiments, namely double knock-down and overexpression in S2 cells. As shown in Fig. 2, we observed significantly decreased *Attacin-luciferase* activity in the double knock-down condition, but the opposite phenotype was not noted in the overexpression analyses. We also did not observe the interaction between Velo and PGRP-LC in our co-immunoprecipitation assay. By these results, it was difficult to position Velo in the IMD pathway. Interestingly, we obtained a stimulating result from our immune cell staining experiment that Velo localizes, both in the nucleus and cytoplasm, but the majority is located in the nucleus (Fig. 3B,C). Indeed, protein sequence annotation database Pantree^66^ predicted that CG10107 (unannotated gene before naming as Velo) is localized in both the nucleus and cytoplasm (ID number: PTN002930416). In the following studies, Berdnik et al. observed in their transgenic flies that a long-form of Velo is localized in the nucleus, proposing that Velo might act as SUMO protease in the nucleus^57^. Dr. Cavalli’s group elegantly discovered that Velo is required for the deSUMOylation of epigenetic repressor Polycomb (Pc) protein and changing the distribution and binding of PcG proteins to their chromatin targeting sites in the nucleus^67^. Interestingly, the *Drosophila* homolog of the yeast SWI2/SFN2 gene, Brahma (Brm), was isolated as a dominant suppressor of Pc mutations^68^ and reported to be a co-activator of *trithorax* group (*trx*G) protein Zeste^69^. This Brahma complex together with a novel nuclear factor Akirin was shown to be required for the transcription of a subset of effector genes in the IMD pathway ^70^.

In mammals, there are at least two different isoforms of SENP7 (the closest homolog to Velo), namely long (SENP7L) and short (SENP7S). SENP7L resides mainly in the nucleus, whereas SENP7S is exclusively localized in the cytosol^71^. SENP7L contains a conserved heterochromatin protein 1 homolog (HP1)-box (PxVxL) motif which determines the mutual recruitment of SENP7 and HP1α to heterochromatin^72,73^. In contrast, SENP7S lacks this HP-1-binding domain, explaining its cytosolic distribution^71^. Interestingly, the short-form of Velo contains one PxVxL motif at the amino acid 89th position, whereas the long-form contains two motifs at the 1068th and 1211th positions. Coherent to this, our immune cell staining result showed that the short-form of Velo has more cytoplasmic diffusion compared to the long-form (Fig. 3C). It is also interesting to note that Studencka et al. reported that HP1 and linker histone is required for the regulation of innate immune gene expression in *C. elegans*^74^. Velo-mediated modulation of chromatin remodeling factors and identification of the cytosolic partners will be one of our future investigations.

Taken together, our data indicated that Velo is a new negative regulator of the IMD pathway both in vitro and in vivo systems. We suspect that due to some technical limitations, we cannot address some critical questions yet, like what are the targets of Velos in the nucleus and/or the cytoplasm, what if nuclear Velo affects chromatin remodeling or probably modulates Akirin-mediated NF-κB signaling^58,70,75,76^, how Velo contributes to SUMOylation status of the IMD pathway, etc. Further analyses will be required to understand the Velo-mediated negative regulation mechanism in the IMD pathway.

## Supplementary Information

Supplementary Information 1.

## Data Availability

The data that support the findings of this study are available from the corresponding author, [A.G.], upon reasonable request.
