# Supplementary material for: Verloren negatively regulates the expression of IMD pathway dependent antimicrobial peptides in Drosophila
Source: Sci Rep. 2021 Jul 30;11:15549. doi: 10.1038/s41598-021-94973-0 (PMC8324896; doi:10.1038/s41598-021-94973-0)

Supplementary information

Verloren negatively regulates the expression of IMD pathway dependent antimicrobial peptides in *Drosophila*

Pragya Prakash[1], Arghyashree Roychowdhury-Sinha[1], Akira Goto[1,2,*]

[1]INSERM, Université de Strasbourg, CNRS, Insect Models of Innate Immunity (M3I; UPR9022), F-67084, Strasbourg, France.

[2]Sino-French Hoffmann Institute, School of Basic Medical Science, Guangzhou Medical University, Guangzhou, 511436, China.

*Corresponding author: goto@unistra.fr (A.G.)

Running title: Negative regulation of the IMD pathway by Verloren.

Supplementary Figure 1.

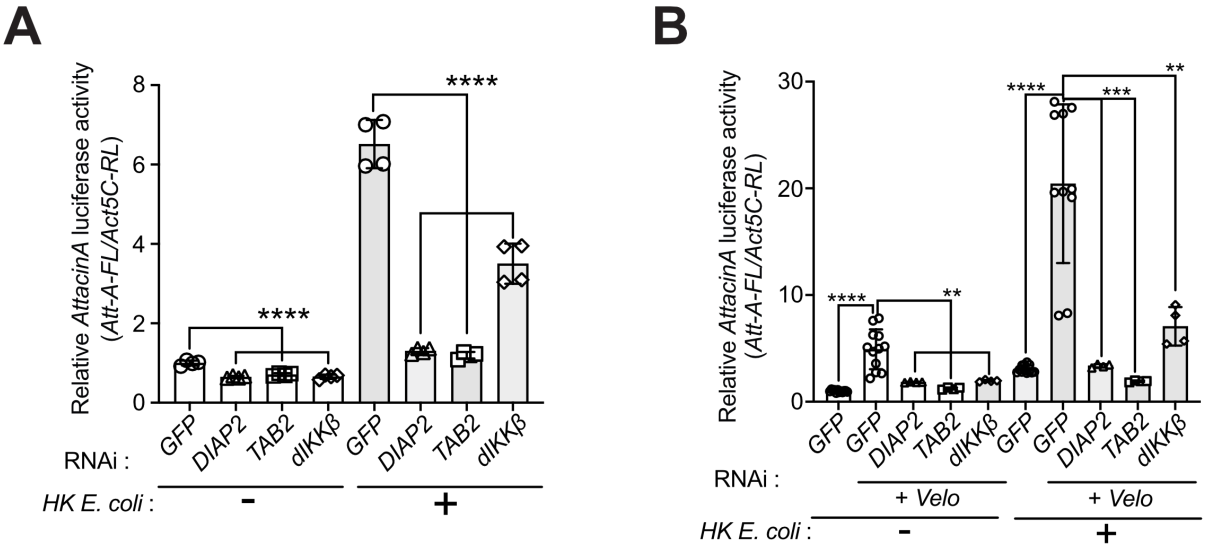

Supplementary Figure 1. Epistatic analyses of Velo in the IMD pathway. (**A,B**) *DIAP2*, *TAB2,* or *dIKKβ*, was knocked down individually (**A**) or together with *Velo* (**B**). After the indicated dsRNA transfection, the activation of the IMD pathway was monitored by *AttacinA-luciferase* activity (*Att-A-FL/Act5C-RL*) in the absence or presence of heat-killed *E. coli* stimulation. The value of *GFP* knock-down control cells was set as 1.

Supplementary Figure 2.

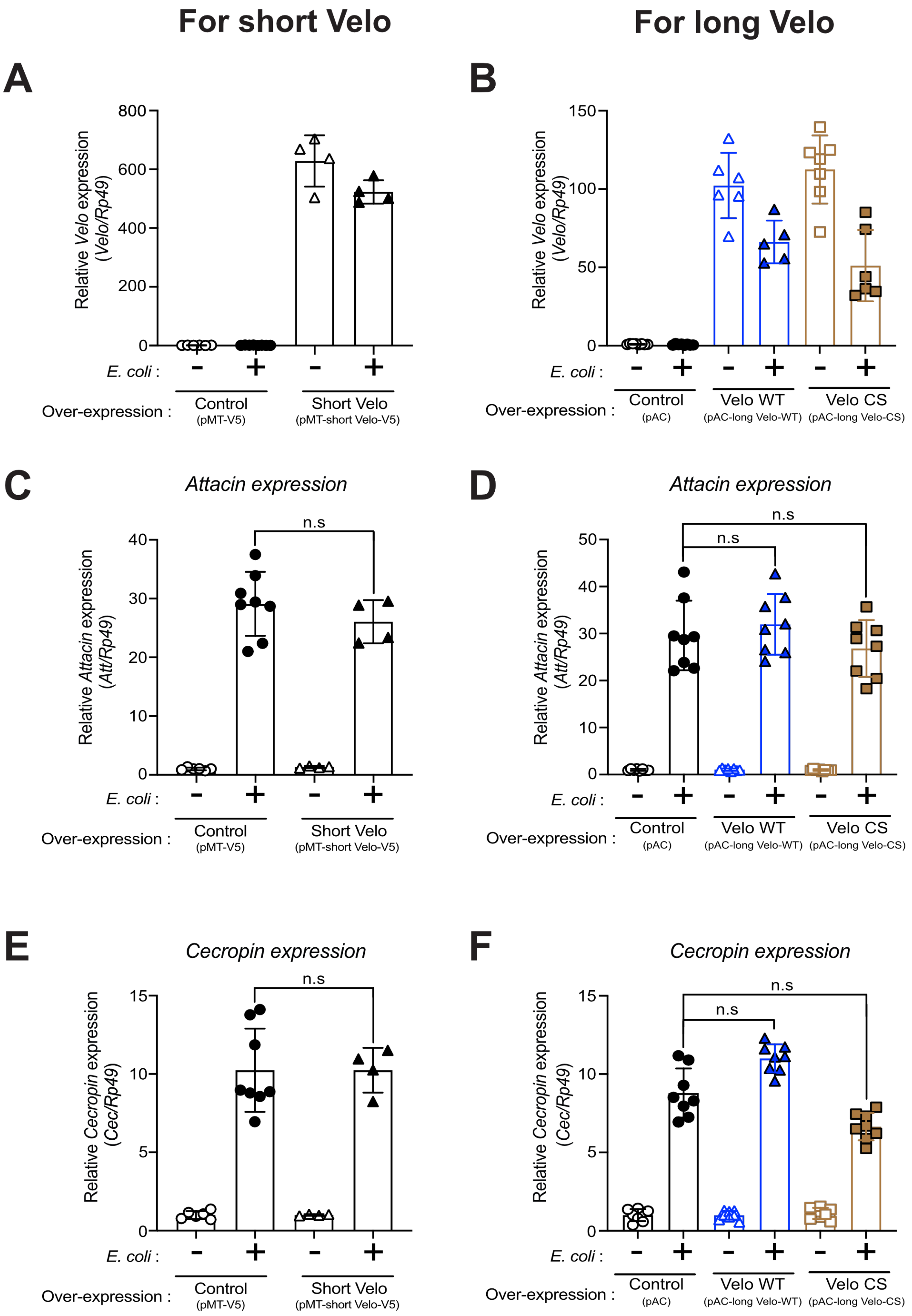

Supplementary Figure 2. Endogenous *Attacin* and *Cecropin* expressions are unaffected in Velo overexpressed S2 cells. The plasmid encoding for a short-form

(Short Velo) **(A, C, E)** or long-form of Velos (either wild-type "Velo WT" or protease-dead "Velo CS") **(B, D, F)** was transfected into S2 cells, and endogenous expression level of *Velo* **(A,B)**, *Attacin* **(C,D)**, and *Cecropin* **(E,F)** was monitored by RT-qPCR before or after heat-killed *E. coli* stimulation. Expression of the Ribosomal protein 49 (*Rp49*) was used as the internal control for normalization. The data points are collected from at least two independent experiments. Each experiment includes at least two bio-replicates. Student's *t*-test was used for statistical analysis: n.s. indicates statistically non-significant.

Supplementary Figure 3.

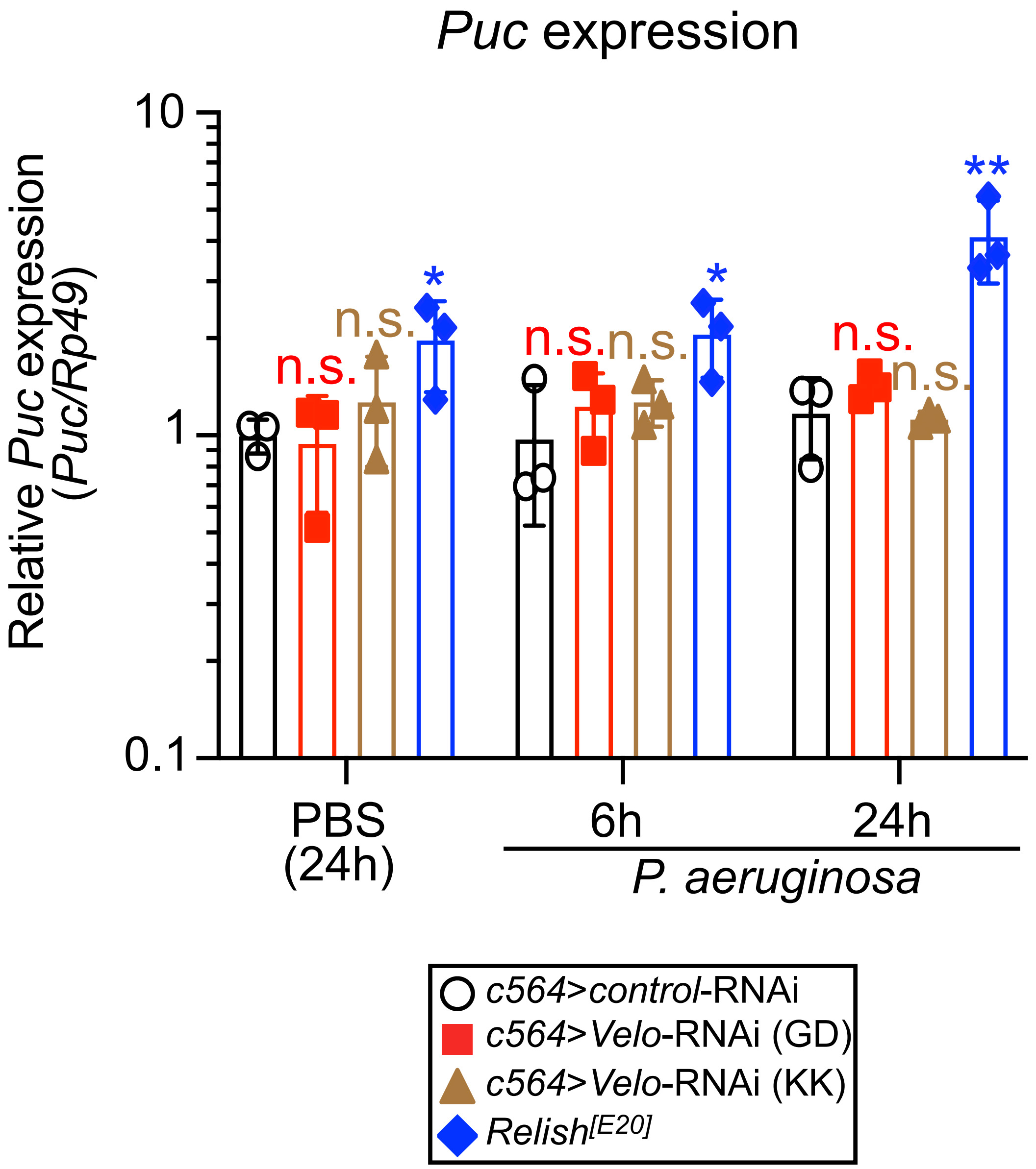

Supplementary Figure 3. Fat body-specific *Velo* knock-down flies did not alter *Puckered* expression to *P. aeruginosa* infection. Two independent UAS-*Velo*-RNAi flies designated as (GD) and (KK) were crossed with *c564-GAL4* driver to generate the fat body-specific *Velo* knock-down flies (*c564>Velo*-RNAi). *c564>Velo*-RNAi flies were pricked by *P. aeruginosa* and the expression of *puckered* was monitored at 6h and 24h post-infection. *c564>control*-RNAi flies were used as a control. Expression of the *Ribosomal protein 49* (*Rp49*) was used as an internal control for

normalization. Control PBS buffer pricked flies were used as control. The data represent the mean and standard error of three biological replicates, and one data point represents a pool of 6-8 flies. The difference between *control*-RNAi and each *Velo*-RNAi is statistically significant (student's *t*-test: *p<0.05, **p<0.01. n.s. indicates statistically non-significant).

Supplementary Table 1.

Primer sequences for RT-qPCR		
**Gene**	**Forward primer, 5'- sequence -3'**	**Reverse primer, 5'- sequence -3'**
RP49	GCCGCTTCAAGGGACAGTATCT	AAACGCGGTTCTGCATGAG
Velo	CTCAAGACGTGTGCCGAATA	CGTCTTGAAGCCGGTATGTT
Diptericin	GCTGCGCAATCGCTTCTACT	TGGTGGAGTGGGCTTCATG
Attacin	GGCCCATGCCAATTTATTC	AGCAAAGACCTTGGCATCC
Metchnikowin	CGTCACCAGGGACCCATTT	CCGGTCTTGGTTGGTTAGGA
Cecropin	ACGCGTTGGTCAGCACACT	ACATTGGCGGCTTGTTGAG
Drosomycin	CGTGAGAACCTTTTCCAATATGATG	TCCCAGGACCACCAGCAT
Puckered	AAATACCTGCCAGCGATACG	CGCAAAGGAACTTGGAAGAG

Full length gel image for Figure 2

IP by anti-V5 (PGRP-LC(TM+Intra)-V5) and WB by anti-HA (Velo-HA).

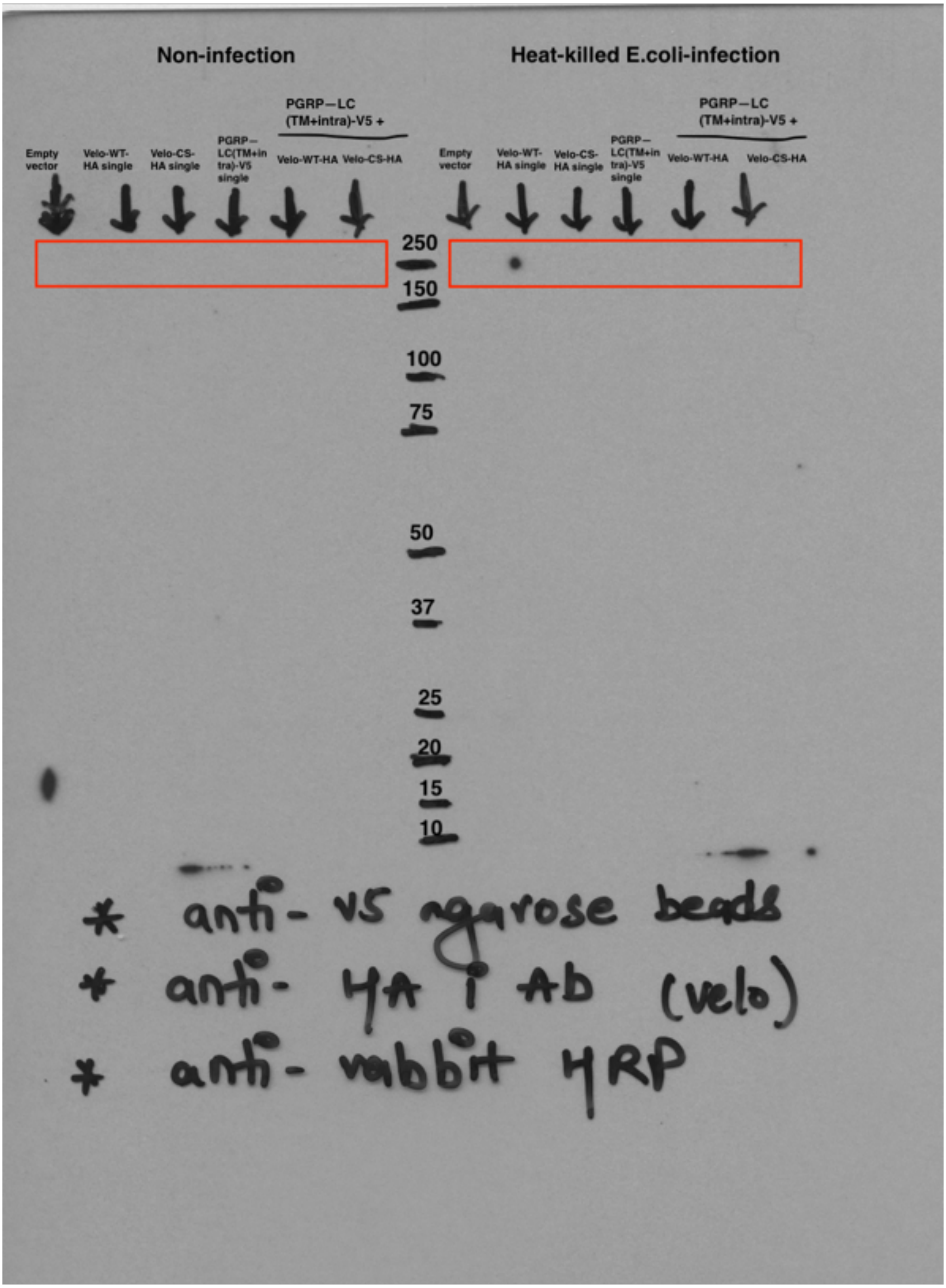

Full length gel image for Figure 2

IP by anti-V5 (PGRP-LC(TM+Intra)-V5) and WB by anti-V5 (PGRP-LC(TM+Intra)-V5). – Due to reverse direction of the X-ray film, the lane order is opposite from the above.

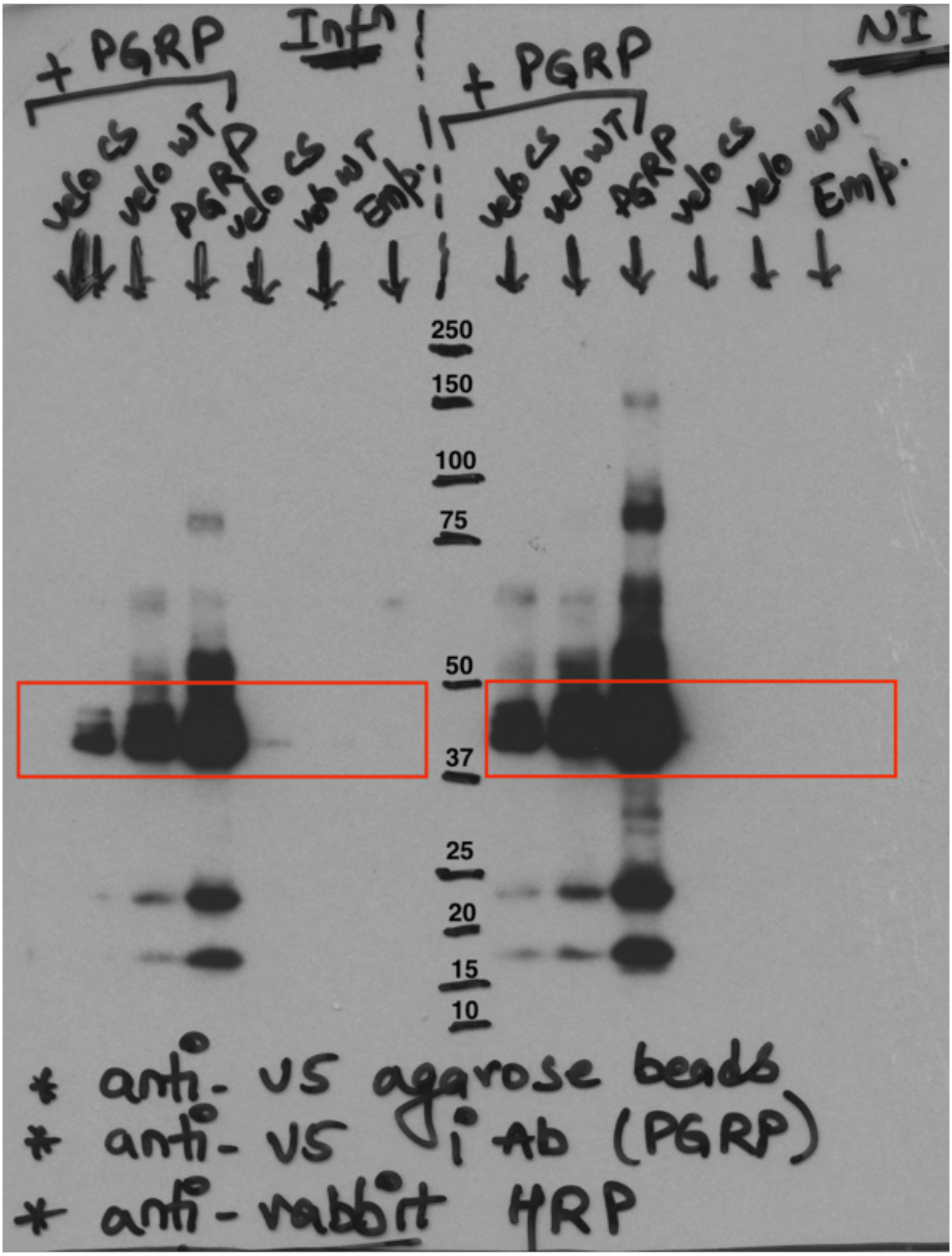

Full length gel image for Figure 2

WB by anti-HA (Velo-HA) for the cell lysate.

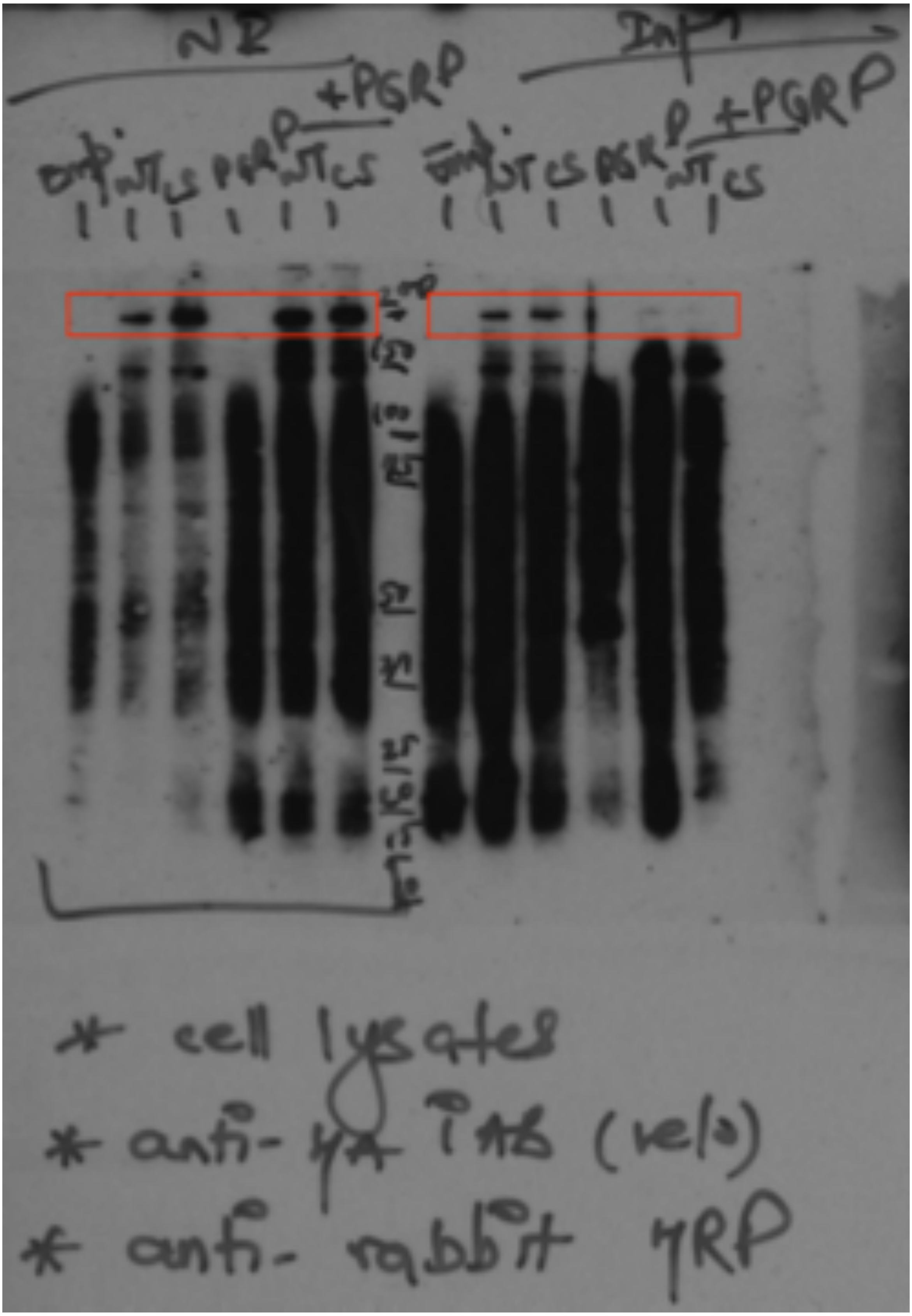

Full length gel image for Figure 2

WB by anti-V5 (PGRP-LC(TM+Intra)-V5) for the cell lysate.

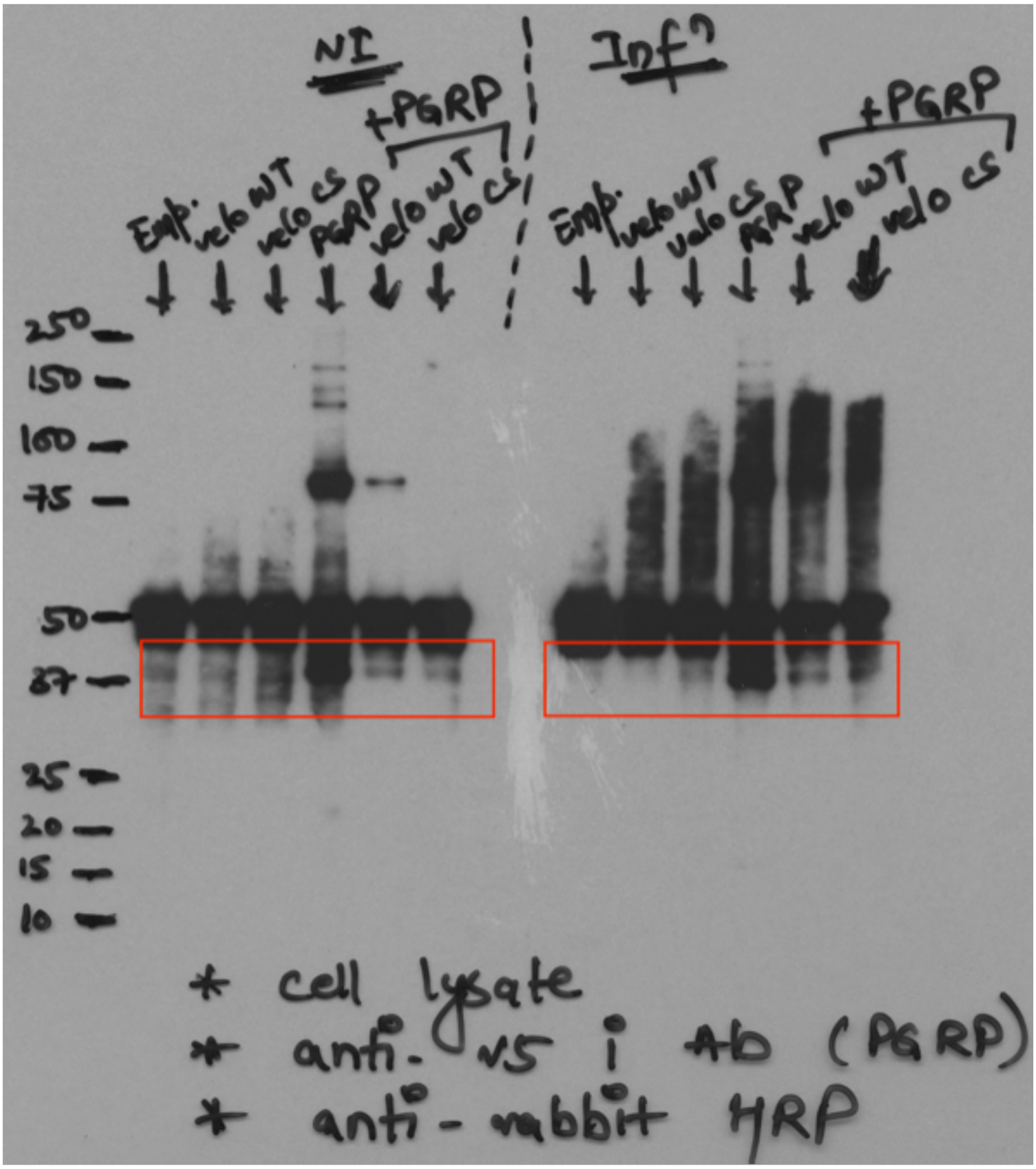

Full length gel image for Figure 2

WB by anti-actin for the cell lysate.

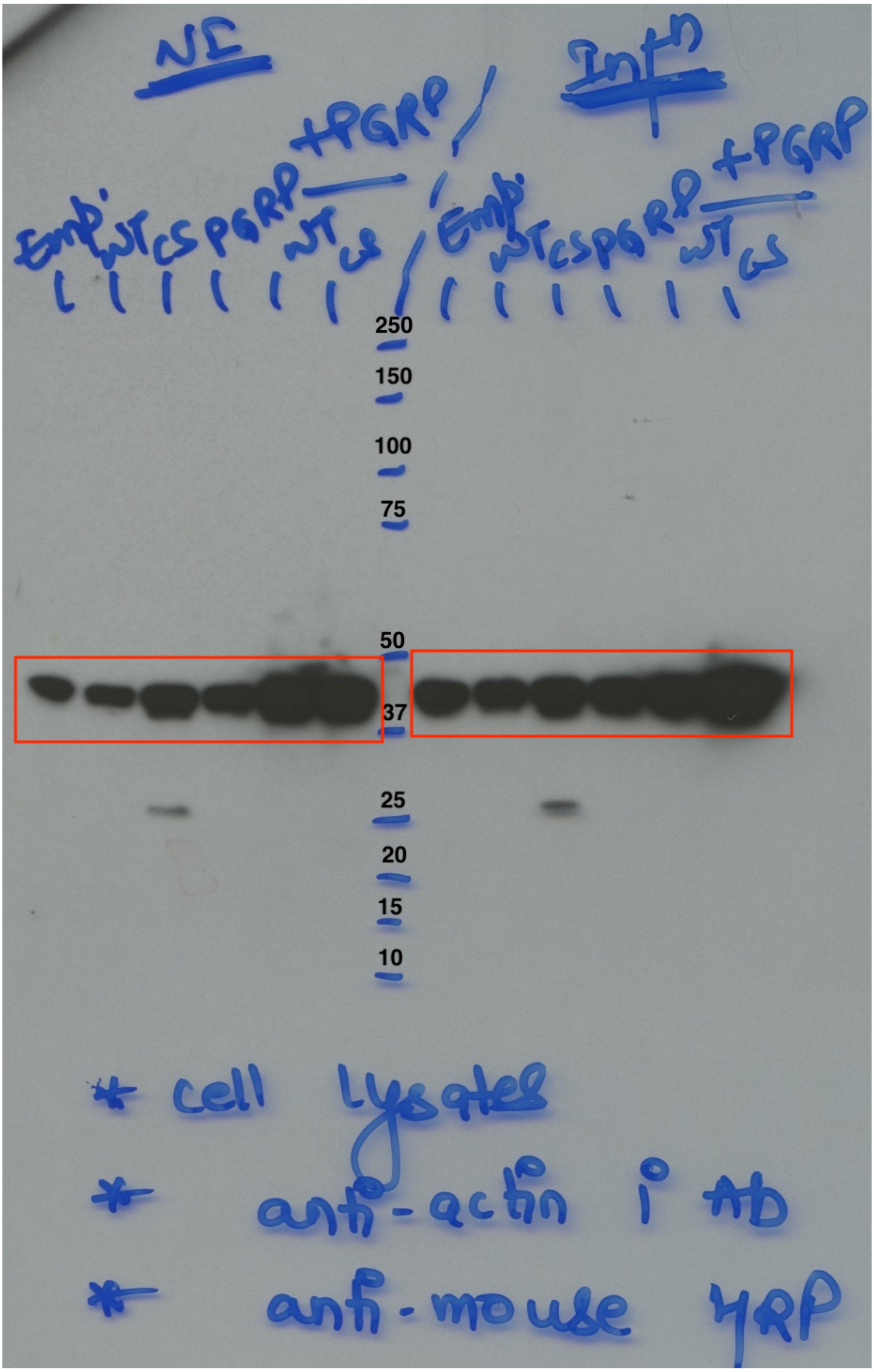

Full length gel image for Figure 3

WB by anti-V5 (Short-Velo-V5) and anti-HA (Long-Velo-HA) for the cell lysate.

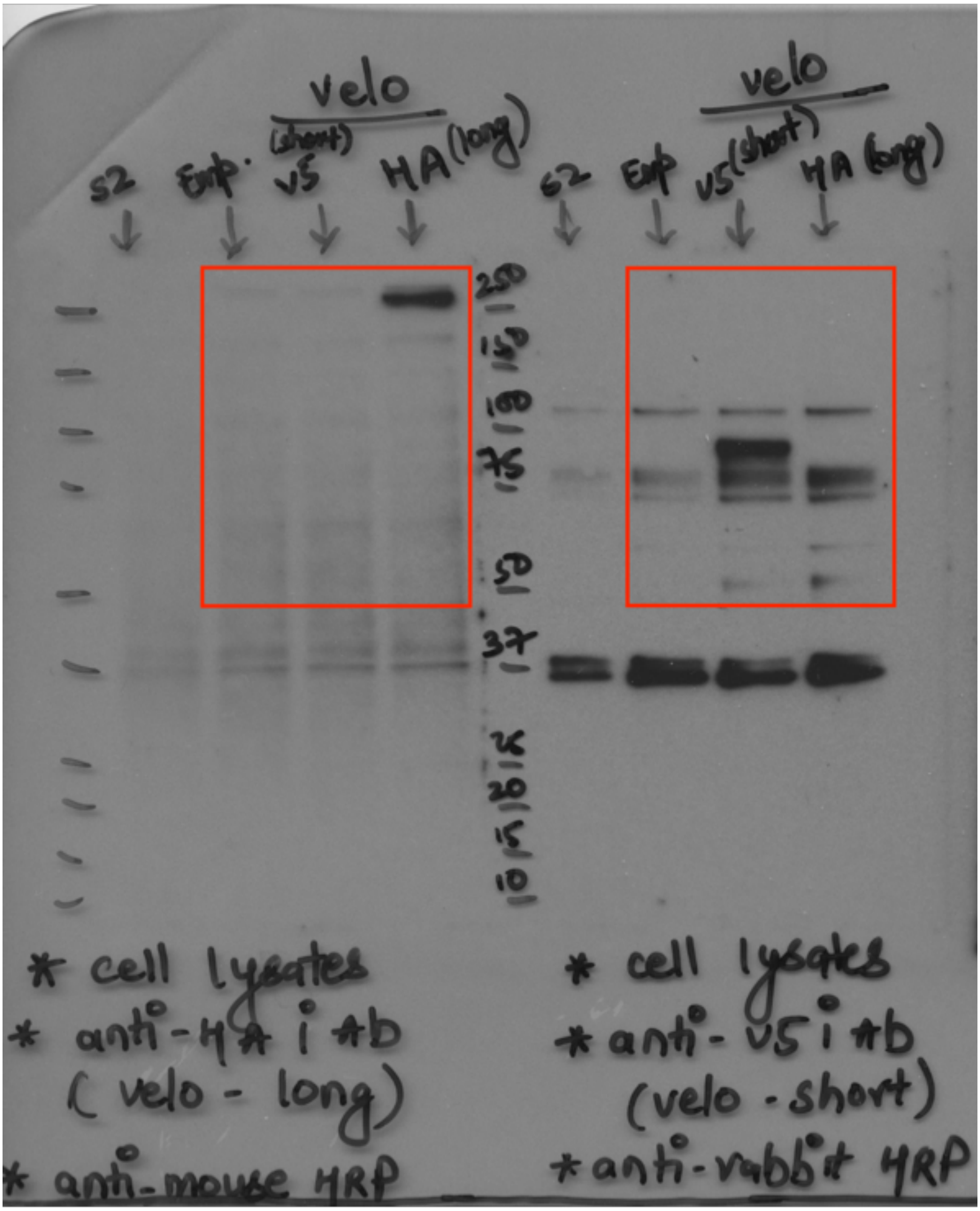

Full length gel image for Figure 3

WB by anti-actin for the cell lysate.

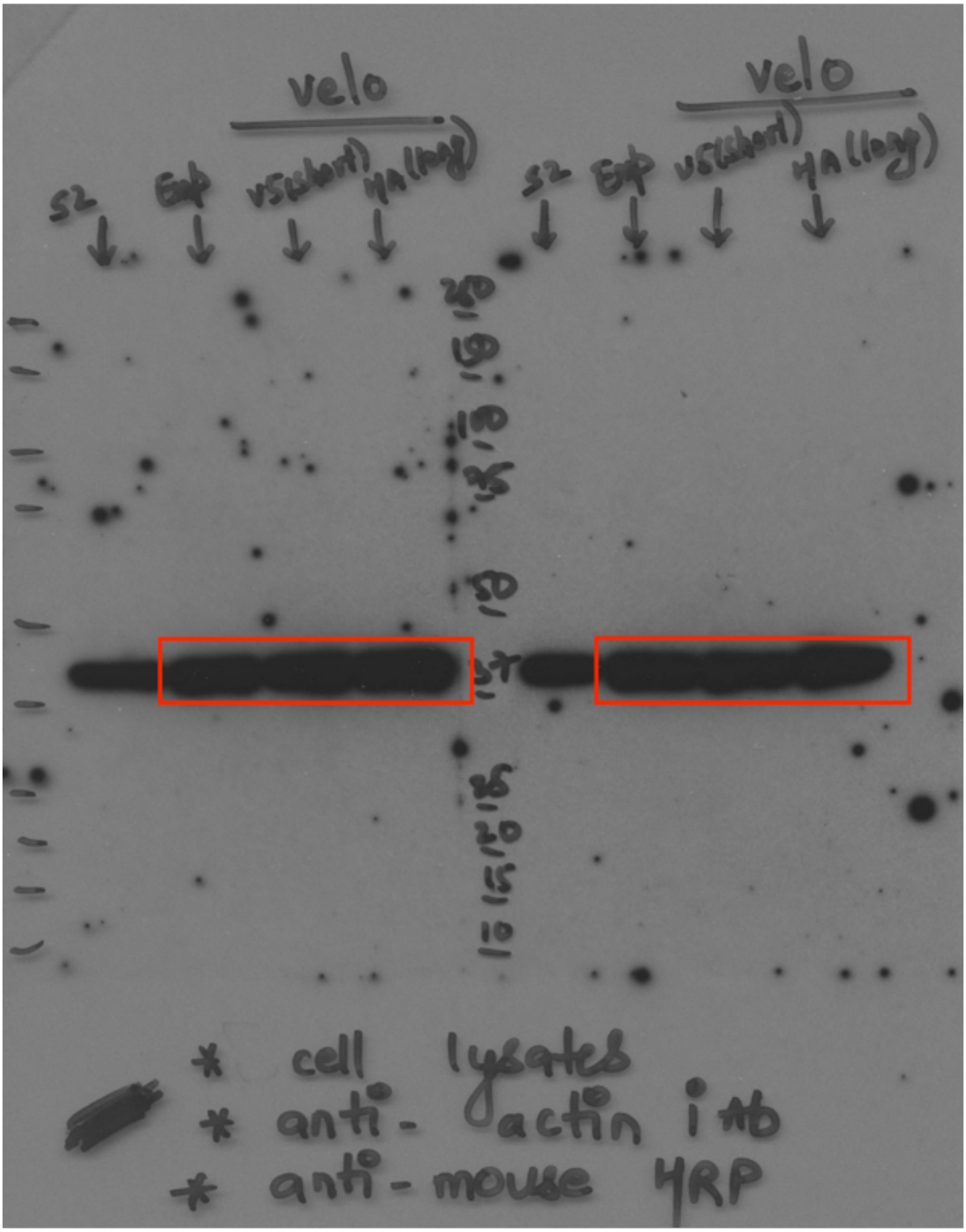

Supplement: Supplementary file 1 — Supplementary Information 1. [file 41598_2021_94973_MOESM1_ESM.pdf]
